# Use of drones to study the behavior of buffaloes in a production system in the Eastern Amazon

**DOI:** 10.29374/2527-2179.bjvm003325

**Published:** 2025-07-25

**Authors:** Maria Angélica Damasceno Rocha, Frederico Ozanan Barros Monteiro

**Affiliations:** 1 Programa de Pós-graduação em Saúde e Produção Animal, Universidade Federal Rural da Amazônia, Belém, PA, Brazil.

**Keywords:** drone, animal behaviour, Murrah, drone, comportamento animal, Murrah

## Abstract

This study aimed to analyze the use of drones for behavioral monitoring of Murrah buffaloes in extensive and intensive production systems in Amapá, Brazil, contributing to the implementation of more accurate and sustainable breeding practices and reducing the need for labor. Ethograms were constructed at a height of 15 m, allowing safe and noninvasive identification of behaviors. The distribution of the data was analyzed for normality using the Shapiro–Wilk test (W = 0.803; p < 0.05) and homoscedasticity (F = 0.345; p = 0.558), which was shown to be homogeneous. Animal reactivity was evaluated using Spearman’s correlation coefficient, and the environmental effects on the response variables were evaluated using PERMANOVA. PCoA was used to explore the spatial distribution of the data. After 104 h of image storage, 17 behavioral types were identified. These results validated the use of the DJI Mini 2 drone for minimally invasive, effective, and low-cost aerial monitoring. The reactivity of the buffaloes to the drone decreased with increasing altitude, with 15 m being ideal for monitoring because it minimized stress and behavioral changes. Confined buffaloes (area 2) showed greater reactivity and spent more time in alert and tense states than buffaloes in pasture (area 1), which showed less reactivity due to more environmental stimuli. Multivariate analysis and PERMANOVA confirmed significant differences between the areas, with area 1 showing greater behavioral diversity (12 types) than area 2 (eight types).

## Resumo

Este estudo teve como objetivo analisar o uso de drones para monitoramento comportamental de búfalos Murrah em sistemas de produção extensiva e intensiva no Amapá, Brasil, contribuindo para a implementação de práticas de criação mais precisas e sustentáveis e reduzindo a necessidade de mão de obra. Etogramas foram construídos a uma altura de 15 m, permitindo a identificação segura e não invasiva dos comportamentos. A distribuição dos dados foi analisada quanto à normalidade usando o teste de Shapiro-Wilk (W = 0,803, p < 0,05) e homocedasticidade (F = 0,345; p = 0,558), que se mostrou homogênea. A reatividade animal foi avaliada usando o coeficiente de correlação de Spearman, e os efeitos ambientais nas variáveis de resposta foram avaliados usando PERMANOVA. PCoA foi usado para explorar a distribuição espacial dos dados. Após 104 h de armazenamento das imagens, 17 tipos comportamentais foram identificados. Esses resultados validaram o uso do drone DJI Mini 2 para monitoramento aéreo minimamente invasivo, eficaz e de baixo custo. A reatividade dos búfalos ao drone diminuiu com o aumento da altitude, sendo 15 m o ideal para monitoramento, pois minimiza o estresse e as mudanças comportamentais. Búfalos confinados (área 2) apresentaram maior reatividade e passaram mais tempo em estado de alerta e tensão do que búfalos em pastagem (área 1), que apresentaram menor reatividade devido a mais estímulos ambientais. A análise multivariada e a PERMANOVA confirmaram diferenças significativas entre as áreas, com a área 1 apresentando maior diversidade comportamental (12 tipos) do que a área 2 (oito tipos).

## Introduction

Buffalo (*Bubalus bubalis*, Linnaeus, 1758) is a ruminant mammal that exhibits adaptability to various soil and climate conditions, showing satisfactory performance in subtropical and tropical regions of the world (Cockrill, 1984; Martínez et al., 2023). Known for its docility, buffaloes are highly regarded by breeders and researchers as an asset for milk, meat, rural tourism, and leather production (Damasceno et al., 2010). In Brazil, the main buffalo breeds are the Carabao, Mediterranean, Jafarabadi, and Murrah.

Buffaloes are adaptable to different ecosystems, with a significant presence in regions with favorable conditions, such as floodplains. Buffalo farming has gained increasing importance because of its economic contributions and the global growth of herds (Instituto Brasileiro de Geografia e Estatística, 2023). This expansion is supported by development programs and the adoption of technological innovations that have transformed animal production, enhancing efficiency and sustainability in the sector.

Among technological advancements, precision livestock farming has become increasingly prevalent in animal production systems, promoting sustainability and operational efficiency. These technologies optimize resource use, reduce water and energy consumption, improve product quality, facilitate individualized monitoring, and significantly contribute to animal welfare (Aquilani et al., 2022; Empresa Brasileira de Pesquisa Agropecuária, 2024; Duffy et al., 2020).

Among the most promising innovations in this area is the use of drones, which have expanded the capabilities of observation and monitoring in livestock management. Drones provide valuable data on production, behavior, and physiological conditions that are essential for animal health, productivity, and welfare (Bernardi et al., 2017). Furthermore, they enable the early detection of health issues, identification of abnormal behaviors, and monitoring of environmental stressors, allowing for prompt interventions to prevent disease outbreaks and improve animal welfare (DJI, 2024; Schad and Fischer, 2022).

Despite the promising potential of drones, further studies are necessary to validate their effectiveness and safety for non-invasive aerial monitoring. Research gaps remain regarding the behavioral effects of drone flights on livestock species, particularly buffaloes. Limited information exists on the effect of drones on buffalo behavior during key activities, such as grazing, social interactions, rumination, resting, feeding, and nursing.

This knowledge gap presents an opportunity for further research aimed at improving livestock production, environmental management, health, and welfare. Strategic application of drones in animal management can transform livestock practices and foster sustainable and efficient production systems.

Therefore, this study aimed to assess the use of drones in the behavioral monitoring of buffaloes, contributing to the development of more precise and sustainable practices in buffalo farming. These findings provide valuable insights for producers and researchers, promoting the adoption of noninvasive technologies that enhance animal health, welfare, and productivity.

## Material and methods

### Study sites

The study was approved by the Ethics Committee on the Use of Animals (CEUA) of the Universidade Federal Rural da Amazônia (CEUA Protocol No. 9709030324/ID 000674). Authorization for each flight was obtained from the Department of Airspace Control in accordance with the instructions outlined in the Aeronautical Information Circular (AIC-N21/10). A Certificate of Registration of Unmanned Aircraft for Non-Recreational Use, issued by ANAC, was obtained with registration number PP-865022546. This study was conducted in two different production systems: areas 1 (extensive breeding) and 2 (intensive breeding).

**Figure 1:**
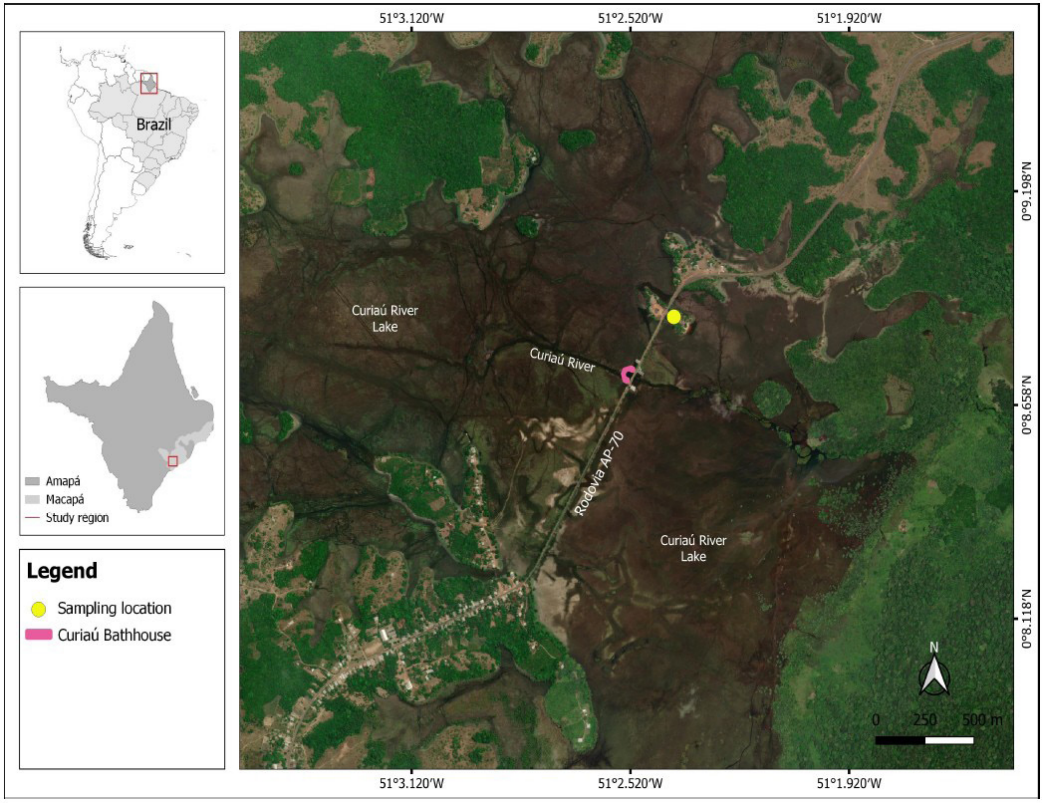
Area 1: Retiro São Francisco, a family-owned quilombola property located in Quilombo do Curiaú, Macapá, AP, featuring an extensive production system. The research sampling location is marked with a yellow dot at coordinates Latitude 0°8'53"N and Longitude 51°2'24"W.

Area 1 was characterized as a family quilombola property called Retiro São Francisco do Quilombo do Curiaú (Macapá – AP) (Lat.; 8º53' N, Long.; 51º24' W), with an extensive production system of Murrah buffaloes (Figure 1). The herd is domesticated and different from animals in nearby properties. Therefore, no fences were constructed to define these properties. The property has 80 animals, including one adult male, 12 male calves, and 67 females, distributed approximately 300 ha.

**Figure 2:**
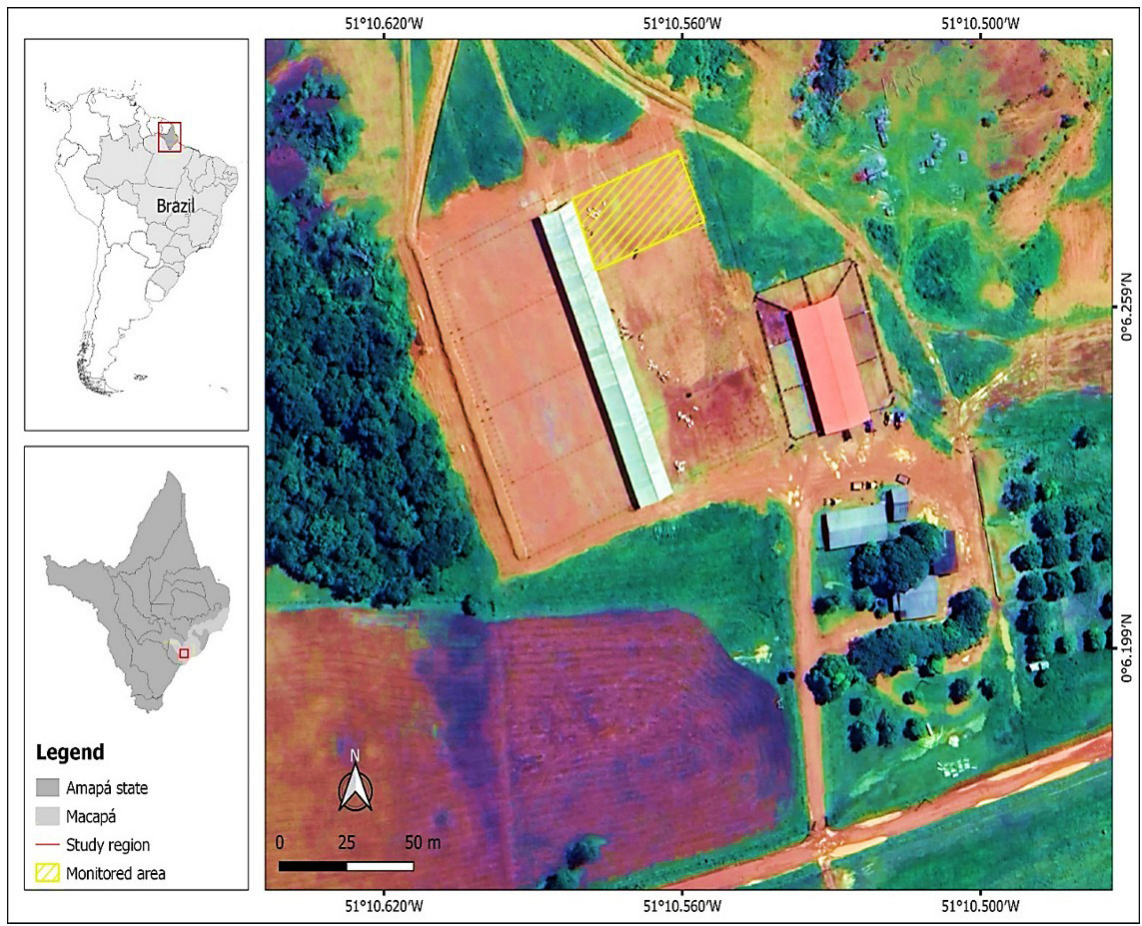
Area 2: Agromix Farm, located in Macapá, AP, is characterized by an intensive production system. Plot 1 (yellow) indicates the focal point of the observations, with coordinates at latitude 0°6'14"N and longitude 51°10'31"W.

Area 2 is characterized by an intensive production system (confinement). Agromix Farm (Macapá – AP) (Lat.; 6º14' N, Long.; 51º10' W) covers 400 ha (Figure 2). Here, it stands out for its potential in raising buffaloes for slaughter. It has 180 Murrah buffaloes, distributed in paddocks with free access to water and bulky and concentrated feed provided in the trough twice daily. This research includes 10 animals; one adult male and nine females of reproductive age.

### Sample collection and processing

The research images are collected through flyovers using a Mini 2 drone from DJI as the observation platform, equipped with a 4 K camera sensor. This quadcopter captures videos at a 4K resolution (4096 × 2160 pixels per frame) and has a flight autonomy of 31 min. Its weight (including the battery and propellers) is 242 g, and it comprises three rechargeable batteries. It was chosen for this study because it is a reference drone for producing low noise levels compared with other drones with similar characteristics. Additionally, it is compact and neutral in color.

The captured images were recorded from orthogonal (vertical, at 90º), capturing detailed portions of the animal, and oblique perspectives, showing the animal from different angles for general characterization of the animal and its environment. The images were transmitted and processed using the DJI Fly software installed on a mobile device and stored on a 128 GB SD card.

**Figure 3:**
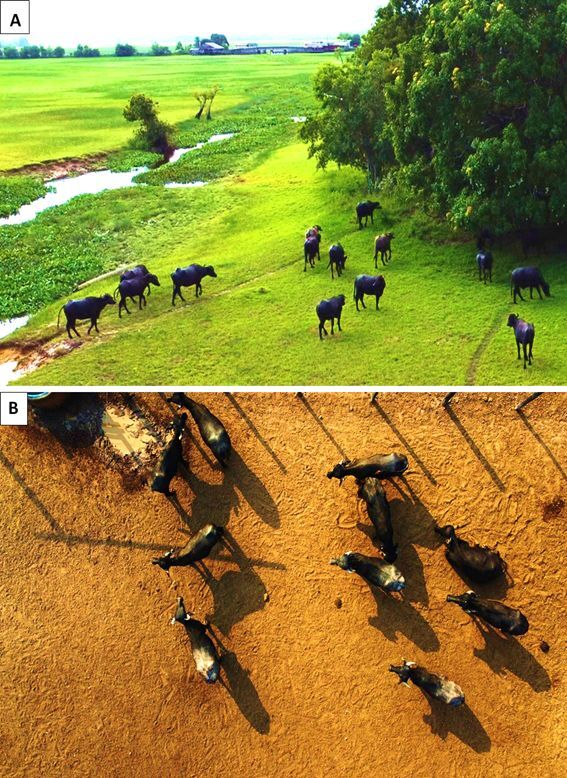
(A) Aerial image captured by a drone at Retiro São Francisco (Quilombo do Curiaú, AP, Brazil), showing extensive buffalo farming. (B) Aerial image captured by a drone at Fazenda Agromix (Macapá, AP, Brazil), providing an aerial perspective of the confinement area.

Aerial monitoring resulted in a significant dataset comprising 104 h of stored images. Data were collected through aerial monitoring at the research control points. In area 1, the control point corresponded to 10,600 m^2^ and in area 2, the control point was Picket 1 (Figure 3).

The buffaloes are monitored in two stages (1 and 2). Stage 1 was conducted to determine the optimal altitude for the drone in relation to the animals, ensuring that there was no significant interference with their behavior during the flights. Therefore, the Drone Altitude Test was applied, in which the duration of the animal’s response time to the presence and noise generated by the equipment (noise from the propellers and engine) was recorded. This test was conducted for 16 h of footage (8 h in each area). For this stage, only a single flight line was conducted during monitoring.

An initial departure height of 20 m was established for the transect to avoid negative behavioral responses from the animals and ensure equipment safety. The height scales defined for the test were 20, 15, 10, 5, 3, and 2 m. At each height, the drone monitored the animals for 10 min. This pattern was repeated twice daily, once in the morning (9:00–10:50 a.m.) and afternoon (5:00 p.m. to 6:50 p.m.), for four consecutive days. If the closest group of buffaloes was randomly selected, the observations focused on the animals that first noticed the presence of the drone. This was the focal animal used for testing.

The test initially took place in area 1 with the drone departing from the outer right edge of the AP 070 highway (starting point) at a height of 20 m and flight speed of 10 km/h. The distances covered varied as follows; 115, 113, 98, and 121 m on days 1, 2, 3, and 4, respectively. The same procedure was conducted in area 2, in which the animals were confined, with the drone starting 20 m from the initial point. The drone maintained a flight speed of 10 km/h and covered a fixed distance of 62 m on all flights to paddock 1. Although the manufacturer recommends a flight autonomy of 31 min for safety reasons, this study used a margin of 20 min per battery. In total, 2 h was used daily (1 h in the morning and afternoon/evening).

Stage 2 involves the construction of two ethograms, one for each research area. Using high-resolution images, the frequency of observed behaviors was identified, analyzed, and quantified. Monitoring during this stage was conducted through several flight lines within the control point of each area for 20 min per battery with a 10-min interval for each replacement. The observations took place in the morning (09:00–10:10) and afternoon/evening (17:20–18:30), totaling 2 h daily. In total, 88 h of images were collected (44 h in each area).

For both ethograms and behavioral analyses, observations were made by groups of animals.

### Statistical analysis

Data distribution was tested for normality using the Shapiro–Wilk test (RVAideMemoire package) and showed a non-normal distribution (W = 0.803; p < 0.05) Herve (2023). Homoscedasticity was assessed using Levene’s test (car package), which showed homogeneity (F = 0.345; p = 0.558) Fox & Weisberg (2019). Spearman’s correlation was applied (cor. test function in R) to evaluate the effects of drone height on animal reactivity. A correlation graph was constructed using the ggplot2 package Wickham ( 2016). To assess the impact of the environment (areas 1 and 2) on response duration, PERMANOVA was applied using the response time matrix for each altitude (2, 3, 5, and 10 m), transformed with the (sqrt + 1) function (vegan package) Oksanen, Blanhet, Friendly, Kindt, Legendre (2008). Principal coordinate analysis (PCoA) was used to explore the spatial distribution of the data. The PCoA and PERMANOVA employed the Euclidean distance method. Vector adjustments in the ordination were performed with the “envfit” function (vegan package), using 999 permutations for empirical significance. Data were organized in Microsoft Office Excel 2019, and all analyses were performed using the R Core Team R Core Team (2021) .

## Results

**Figure 4:**
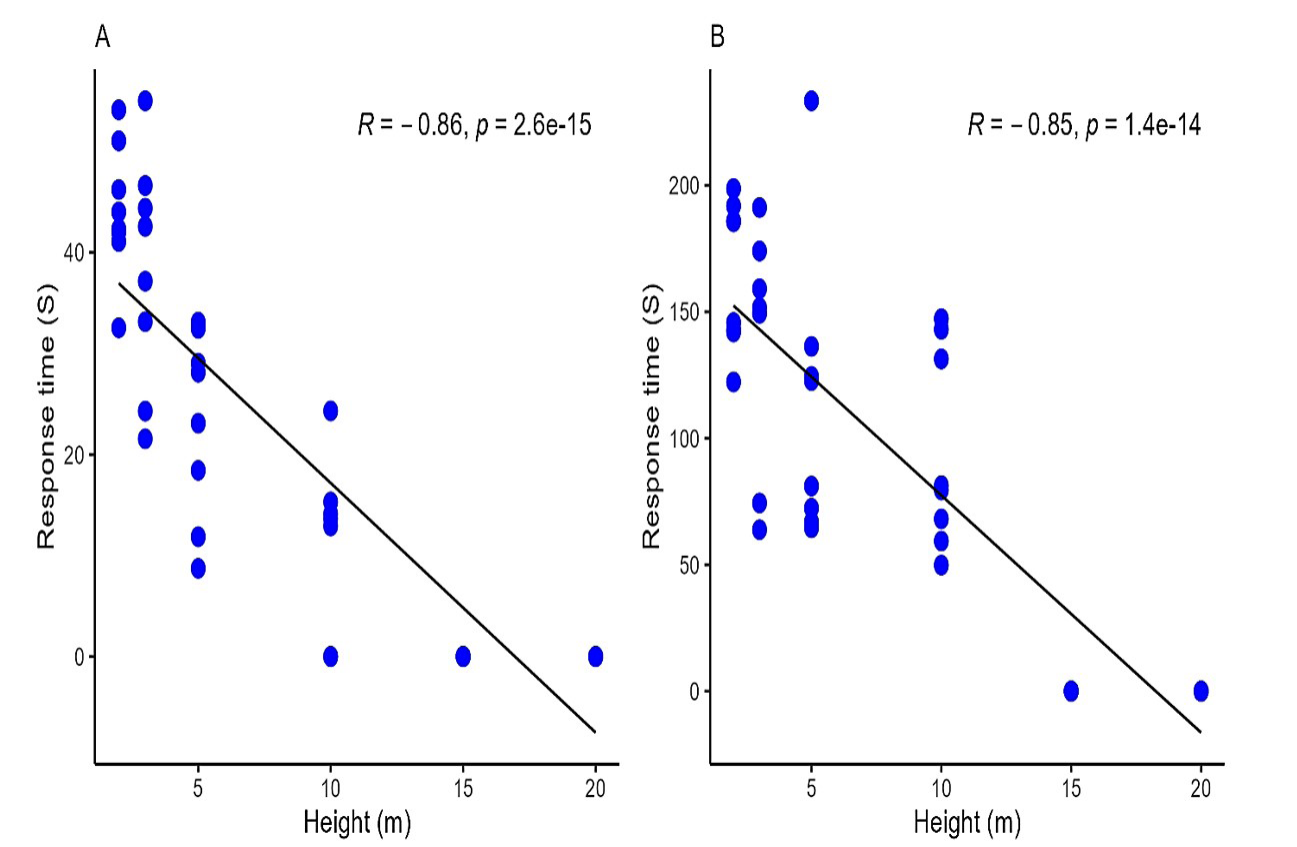
(A) Spearman correlation for Area 1 and (B) Area 2, based on the duration of the response to the drone (in seconds) and the height (in meters).

During the drone altitude test, the relationship between two variables (Figure 4) was observed, analyzed, and displayed in Spearman’s correlation graphs; the duration of the response to the drone (animal reactivity), measured in s, and height of the drone, measured in m.

The blue dots represent the animal’s reactivity to drone flights at altitudes of 20, 15, 10, 5, 3, and 2 m. The variables behave inversely in both graphs, maintaining a negative correlation. As the drone’s altitude increases, the animal shows less reactivity (shorter response time to the drone). Conversely, if the drone’s height decreases, the animal’s reactivity increases (longer response time to the drone). In graph A (area 1), the altitude with the longest duration of reactivity to the drone was at 3 m, with a response time of 55.02 s. In graph B (area 2), the altitude with the longest duration of reactivity to the drone was at 5 m, with a response time of 233.43 s. Particularly, confined animals are more reactive and spend much more time in an alert and/or tense state with the drone than animals in pasture.

According to our results, this temperament can be visually understood through the body postures performed by the buffaloes, which are generally characterized by raising and lowering the head in an alert position, sniffing with a snout to identify the object (drone), and vigorous tail swinging. These behaviors reflected a tendency toward an alert and/or tense state. This is similar to the description by Paranhos da Costa and Magalhães (2015), in which an alert and/or tense animal exhibited abrupt movements of the tail, ears, and head.

**Figure 5:**
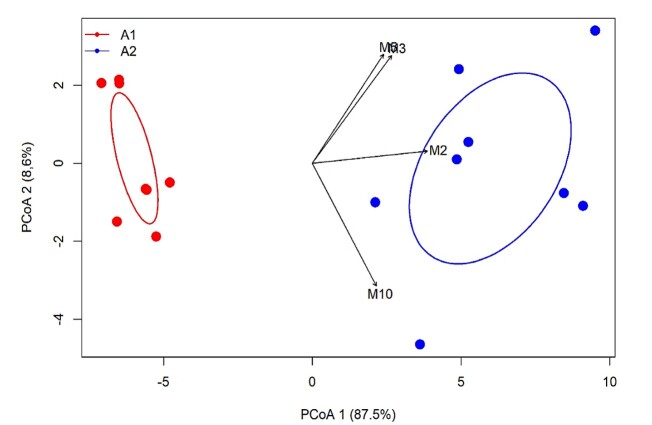
Principal Coordinates Analysis (PCoA) based on the Euclidean distance matrix for drone overflight time data at different altitudes in Areas 1 (A1) and 2 (A2). The percentage of variation explained by the principal coordinates is indicated on the axes.

The Spearman correlation graph shows that in both monitored environments, there was a considerable decrease in reactivity to the drone at a height of 10 m. At 15 and 20 m, the animals either did not perceive or express any changes in their behavior (reaction time = 0), continuing their activities normally in the pasture (area 1) and paddock 1 (area 2). This indicated a positive response to the presence of drones at these altitudes. In PCoA, four variables were considered; M2, M3, M5, and M10 (flight heights), making the analysis multivariate. Excluding M15 and M20, which showed no response, the data resulted in a 2D graph with two axes (PCoA1 and PCoA2) corresponding to areas 1 and 2, respectively. The analysis revealed that animals responded differently to the duration of reactivity in the two environments (Figure 5).

PERMANOVA revealed that the study areas (A1 and A2) influenced the animals’ response duration (F = 55.37; R^2^ = 0.798; p = 0.001). Axes 1 and 2 of the PCoA explained 96.1% of the data variation. The graph clearly shows the formation of two groups, corresponding to the two studied areas (A1 and A2). All sampled altitudes were more strongly associated with area 2, indicating that the longest response durations (reactivity) occurred in this area. The altitude of 2 m was particularly representative and strongly correlated with area 2. The vector adjustment in the ordination, performed by the “envfit” function, shows that all altitudes are significant and influenced the data variation. The combined results from the PERMANOVA and PCoA analysis (Table 1) highlight the application of the approach to both research areas. Statistical significance was set at p < 0.05.

**Table 1:** The envfit test, which used 999 permutations and a Euclidean distance matrix to evaluate the correlation of altitudes (in meters) with the ordination axes (Axis 1 and Axis 2). The R^2^ values indicate the strength of the relationship, while the p-values (all < 0.05) denote statistically significant correlations.

**ALTITUDES (METERS)**	**AXIS 1**	**AXIS 2**	**R2**	**P**
**M2**	0.996	0.080	0.967	0.001
**M3**	0.694	0.719	0.950	0.001
**M5**	0.651	0.758	0.870	0.001
**M10**	0.564	-0.825	0.932	0.001

The average response time of the animals to the presence of the drone in areas 1 and 2 differs. In the average time for axes 1 and 2, applied to the R^2^ test, we found that p-value was < 0.05 at the sampled altitudes (M2, M3, M5, and M10), with a value of 0.001. Therefore, the result is significant. Based on the statistical methods applied, the drone is an effective tool to be used at a safe height of 15 m for monitoring buffaloes, disapproving of its use at lower altitudes. Consequently, the flight protocol was applied for this specific height. This led to the next stage; the construction of ethograms and identification of behaviors.

The images captured by the drone allows the observation of different types of behaviors expressed by the buffaloes in the study areas. In the ethogram of area 1 (Table 2), 12 types of behaviors are identified. In the ethogram of area 2 (Table 3), eight types of behaviors are identified.

**Table 2:** Ethogram of buffaloes monitored from an aerial perspective, Area 1 (extensive breeding).

**Behavioral Unit**	**Descriptors**
**Grazing behavior**	Buffalo moving continuously, searching for food in the pasture with its head tilted, smelling, sniffing, and exploring different areas of the pasture.
**Eating behavior**	Buffalo with its head tilted, alternating between lowering and raising its head, grasping forage with its tongue, and chewing with mechanical movements.
**Rumination (lying down posture)**	Buffalo lying down, resting with its head raised, showing intensive jaw movements, evident salivary secretion, regurgitating partially digested food, and chewing again.
**Rumination (standing posture)**	Buffalo standing at rest, with its head raised, showing intensive jaw movements, evident salivary secretion, regurgitating partially digested food, and chewing again.
**Maternal-filial behavior**	Female buffalo accompanying her calf in the pasture after breastfeeding.
**Breastfeeding**	Calf standing next to its mother, searching for the teats, and sucking to ingest milk.
**Social hierarchy**	Male buffalo in a dominant position, with a proud posture, head held high, and vocalizing.
**Displacement**	Buffalo walking with its head high, showing vigorous movements, a slow and firm gait, coordinated limb motion, vocalizing, and walking along trails in a lined position.
**Group-filial maternal behavior**	Female buffaloes caressing their calves in an integrated manner, while young buffaloes wallow in a mud puddle.
**Socialization**	Young buffaloes in a group, showing sinuous movements, lowering and raising their heads, rubbing against each other, headbutting, and partially immersing in mud.
**Atypical behavior**	Spotted, blind buffalo with its head tilted upwards, sniffing with its snout, reacting to the presence of the drone at 15 meters above.
**Harmonic interspecific relationship**	Buffalo lying in a mud puddle, relaxed, with a bird resting on its back, pecking and capturing parasites with its beak.

**Table 3:** Ethogram of buffaloes monitored from an aerial perspective, Area 2 (intensive breeding).

**Behavioral Unit**	**Descriptors**
**Eating behavior**	Buffalo with its head tilted, alternating between lowering and raising its head, grasping forage with its tongue, and bringing it to its mouth, accompanied by mechanical chewing movements.
**Rumination (lying posture)**	Buffalo lying down, resting with its head raised, showing intense jaw movements, evident salivary secretion, regurgitating partially digested food, and chewing it again.
**Immersed in water**	Buffalo in a lying position, with muscles relaxed, head down touching the ground, body immersed in a mud puddle, and wallowing.
**Social** **hierarchy**	Male buffalo in a proud posture, holding its head high and vocalizing.
**Affective bond**	Buffalo touching another buffalo’s head with its snout, muscles relaxed.
**Approach to water source**	Buffalo walking toward the water source, with its head tilted downward, mouth immersed in the water, drinking by sucking, and alternating with licking.
**Agonistic behavior**	Male buffalo at the trough, threatening females with robust movements, head moving continuously up and down, and vocalizing.
**Prolonged inactivity**	Buffalo in a lying position, showing minimal movements, appearing apathetic, with its head in a neutral position.

**Figure 6:**
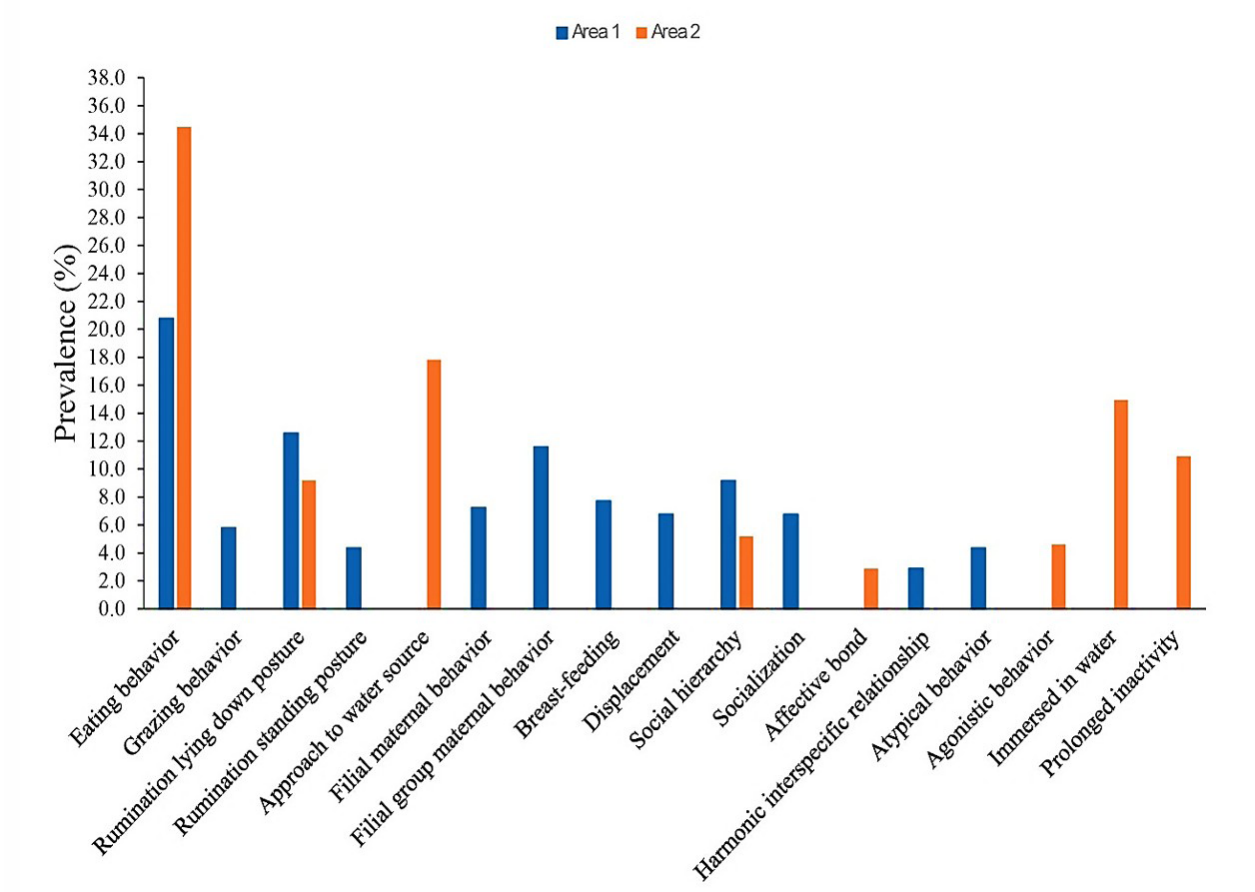
Types of animal behavior expressed as a percentage in relation to the presence of the drone in the two study areas: Area 1 (Retiro São Francisco) and Area 2 (Agromix Farm).

The comparative study of buffalo behavior in the presence of drones reveals distinct behavioral patterns between the two analyzed areas (Figure 6). In area 1, a greater diversity of behaviors is observed. Feeding activities are recorded in 20% of the animals, rumination in a lying position in 12%, and social hierarchical interactions in 10%.

The exclusive behaviors observed in area 1 included grazing, standing rumination, mother-calf relationships (individual and group), nursing, displacement, socialization, and harmonious interspecies interactions. Additionally, atypical behaviors were observed in this region.

Contrastingly, buffaloes in area 2 exhibited a higher frequency of feeding (34%), lower occurrence of lying rumination (9%), and fewer hierarchical interactions (6%). The specific behavioral characteristics of area 2 included approaching water sources, demonstrations of affective bonding, agonistic behaviors, water immersion, and prolonged periods of inactivity.

## Discussion

The flight altitude of the drones is important in the spatial resolution of the captured images and behavior of the animals under observation. Higher altitudes often result in reduced image quality, whereas lower altitudes may cause discomfort or stress in animals (Alencar et al., 2020). Presently, using transects (flight lines) with a flight altitude of 15 m was considered ideal for monitoring buffaloes at control points in the monitored areas (Buckland et al., 2004; Hamilton 2018; Koger et al., 2023; Serpa et al., 2018). These control points were used to validate the information obtained by the drone lenses (Alves, 2023), allowing for the collection of reliable behavioral data without inducing significant behavioral changes in the animals.

The impact of drone flight height on image quality and animal reactivity has been well-documented in the literature. Higher altitudes tend to produce low-resolution images, whereas lower altitudes can cause stress and discomfort (Alencar et al., 2020). Our study confirms that 15 m provides a suitable balance, allowing for effective behavioral monitoring while minimizing disturbances to the natural behavior of buffaloes.

Environmental factors are crucial in shaping animal responses to drone monitoring. Buffaloes in area 1 (extensive) are characterized as raising animals of economic interest exclusively on pasture and making maximum use of natural resources with restricted supplementation (Oliveira et al., 2008). Raising in a quilombola community with frequent human and vehicle activity likely developed a heightened tolerance to various stimuli, including sounds and sights associated with drone flights. This constant exposure to diverse environmental stimuli may have enhanced the resilience of buffaloes to external disturbances, such as drone noise. Contrastingly, buffaloes in area 2 (intensive) are characterized as raising animals of zootechnical interest in a delimited space, with the formation of groups in corrals or paddocks, receiving food and water in a balanced manner (Abreu et al., 2018). If confined to smaller spaces with limited stimuli, they displayed greater reactivity, likely owing to their less varied environments. The lack of environmental enrichment in confined systems may further exacerbate the stress responses. No behavioral enrichment resources, such as forage-searching opportunities, hay balls, or interactive activities, were provided, which limited the buffaloes’ behavioral expression and may have contributed to their increased reactivity. This aligns with the findings of Porto et al. (2018) who noted that reduced behavioral options are linked to higher reactivity and compromised well-being.

Additionally, Oliveira et al. (2021) showed that animals with higher reactivity often experience poorer weight gain and feed efficiency, leading to reduced productivity regarding meat and milk production. According to Arruda et al. (2025) and Khanal et al. (2010), the applicability of drones in confined environments increases productivity by reducing the daily management of herds and labor time. Our observations suggest that 15 m is the optimal drone altitude to avoid inducing stress and facilitate the natural behavioral patterns of buffaloes, promoting a more stable and productive response. Similar to the information from Arruda et al. (2025), drones flying at heights > 5 m did not disturb the cattle.

A comparison of behaviors between the two areas revealed notable differences in behavioral diversity. The buffaloes in area 1 exhibited a broader range of behaviors (207 occurrences), which can be attributed to the more complex and dynamic environment of the extensive system. Conversely, the buffaloes in area 2 demonstrated fewer behavioral expressions (174 occurrences), supporting the idea that restricted environments with limited stimuli can reduce behavioral variety and affect animal welfare. Silva & Ribeiro ( 2021) highlighted that confinement and lack of space are linked to an increase in stereotypical behaviors, which negatively impact animal welfare. Our results suggest that ensuring adequate space and environmental enrichment are crucial for enhancing buffalo welfare in extensive and intensive systems.

Extensive and intensive production systems have advantages and limitations. Buffaloes require optimal conditions, including access to fresh water, adequate nutrition, shade, and sufficient space, to ensure their well-being and productivity. By meeting these requirements, livestock management practices can be improved, thereby benefiting animal welfare and farm productivity.

The use of drones for behavioral monitoring offers substantial potential for large-scale data collection. Drones allow for detailed observations of complex social, feeding, and ecological interactions that are difficult to capture using traditional methods. Consistent with Arruda et al. (2025), DJI mini 2 drones provided detailed images of social behaviors, diseases, fighting interactions, and licking in cattle. This technological approach represents a valuable tool for understanding animal behavior and improving management practices, particularly in extensive and intensive production systems.

## Conclusions

The data obtained validated the use of the DJI Mini 2 drone as a minimally invasive tool for aerial monitoring, offering valuable insights into the behavioral repertoires of buffaloes. Reactivity to the drone decreased with increasing flight altitude, with 15 m identified as the optimal height for monitoring, as it minimized stress responses and behavioral alterations in the animals. Buffaloes housed under confined conditions (area 2) exhibited significantly greater reactivity and spent more time in the alert and tense states than those maintained on pasture (area 1), which demonstrated reduced reactivity, likely because of increased freedom and exposure to environmental stimuli. Multivariate analysis and PERMANOVA confirmed statistically significant behavioral differences between the two areas. Ethogram analyses further revealed greater behavioral diversity in area 1, characterized by 12 distinct behavior types, compared to eight in area 2. These findings underscore the use of drones in facilitating comprehensive spatial analyses and generating high-resolution imagery that supports the management of breeding environments while reducing the need for direct animal handling. Drones provide real-time data that enhance the assessment of key welfare indicators, including environmental comfort, productivity, feeding behavior, morbidity, and mortality.
